# Factors Determining Treatment Success in Children with Drug-Sensitive Tuberculosis in Ethiopia: A Three-Year Retrospective Analysis

**DOI:** 10.4269/ajtmh.19-0816

**Published:** 2020-09-21

**Authors:** Dereje Habte, Yared Tadesse, Dereje Bekele, Genetu Alem, Degu Jerene, Nebiyu Hiruy, Zewdu Gashu, Tadesse Anteneh, Daniel G. Datiko, Yewulsew Kassie, Pedro G. Suarez, Muluken Melese

**Affiliations:** 1 Management Sciences for Health, Addis Ababa, Ethiopia;; 2 Oromia Regional Health Bureau, Addis Ababa, Ethiopia;; 3 Amhara Regional Health Bureau, Baher Dar, Ethiopia;; 4 USAID/Ethiopia, Addis Ababa, Ethiopia;; 5 Management Sciences for Health, Arlington, Virginia

## Abstract

This study in the Amhara and Oromia regions of Ethiopia assessed the outcomes of tuberculosis (TB) treatment among children younger than 15 years. Retrospective data were collected on treatment outcomes and their determinants for children with TB for the cohorts of 2012–2014 enrolled in 40 hospitals and 137 health centers. Chi-square tests, *t*-tests, and logistic regression were used for the analysis. Of 2,557 children registered, 1,218 (47.6%) had clinically diagnosed pulmonary TB, 1,100 (43%) had extrapulmonary TB, and 277 (8.9%) had bacteriologically confirmed TB. Among all cases, 2,503 (97.9%) were newly diagnosed and 178 (7%) were HIV positive. Two-thirds of the children received directly observed treatment (DOT) in health centers and the remaining one-third, in hospitals. The treatment success rate (TSR) was 92.2%, and the death rate was 2.8%. The childhood TSR was high compared with those reported in focal studies in Ethiopia, but no national TSR report for children exists for comparison. Multivariate analysis showed that being older—5–9 years (adjusted odds ratio [AOR], 95% CI: 2.53, 1.30–4.94) and 10–14 years (AOR, 95% CI: 2.71, 1.40–5.26)—enrolled in DOT in a health center (AOR, 95% CI: 2.51, 1.82–3.48), and HIV negative (AOR, 95% CI: 1.77, 1.07–2.93) were predictors of treatment success, whereas underdosing during the intensive phase of treatment (AOR, 95% CI: 0.54, 0.36–0.82) was negatively correlated with treatment success. We recommend more research to determine if intensive monitoring of children with TB, dosage adjustment of anti-TB drugs based on weight changes, and training of health workers on dosage adjustment might improve treatment outcomes.

## INTRODUCTION

Globally, there were an estimated 10 million incident tuberculosis (TB) cases in 2018, of which 11% would be expected to be children younger than 15 years. In 2018, 7 million people with TB were notified worldwide. Childhood TB accounted for 8% of all the notified TB globally. According to the same WHO 2019 report, Ethiopia, one of the 30 high TB-burden countries, notified 113,613 new and relapse cases in all age-groups in 2018. Childhood TB accounted for 10% of all the notified TB cases in Ethiopia, but high proportion of missed cases was reported in the 0–4 age category.^1^ In 2018, Ethiopia also reported a treatment success rate (TSR) of 96% for the cohort of new TB cases of all ages registered in 2017.^1^ Although there are no global data on the TSR for children as a point of comparison, focal studies in Ethiopia have reported a TSR of 78.9% in the northern part of the country,^2^ 63.0% in the south,^3^ and 85.5% in Addis Ababa.^4,5^ In Cape Town, South Africa, a TSR of 89.5% for children younger than 15 years has been reported.^6^

Tuberculosis surveillance data for children in many countries are lacking, and there are few epidemiologic studies.^7^ In Ethiopia, the true burden of childhood TB is unknown because of poor case ascertainment, absence of active case finding, and limited surveillance data. Ethiopia launched a national childhood TB road map in 2015 that prioritized childhood TB interventions to be incorporated into Integrated Management of Neonatal and Childhood Illnesses nationally, although that strategy had been implemented through the Help Ethiopia Address Low TB Performance (HEAL TB) project since 2011. The road map recommends strengthening routine TB screening in clinics and offers guidance on the delivery of quality childhood TB care and treatment services.^8^ The available studies of the epidemiology and treatment outcomes of childhood TB in Ethiopia have focused on a few health facilities or urban settings.^2,5,9,10^

Child-specific performance data are important for understanding the situation in children, exploring the factors associated with program success, and fostering evidence-based decision-making at the policy, planning, and program implementation levels. However, the national health management information system routinely reports aggregated treatment outcomes for all age-groups. Because evidence on the treatment outcomes of children who start treatment for TB in Ethiopia is limited, this study aims to assess the childhood TB TSRs and associated factors in two major agrarian regions of Ethiopia.

## MATERIALS AND METHODS

### The setting.

Data on childhood TB treatment outcomes were collected in the Amhara and Oromia regions of Ethiopia, which, with a total population of 55 million, account for 60% of the country’s population.^11^ In the two regions, there were 121 public hospitals and 2,195 health centers providing TB prevention, control, and treatment services (Regional Health Bureaus, unpublished report, 2017). In this study, 40 hospitals and 137 health centers with high TB patient loads (a minimum of 10 childhood drug-sensitive TB cases per year) were included. Children with multidrug-resistant TB (MDR-TB) were excluded because they were treated in selected MDR-TB centers. The HEAL TB project, funded by the U.S. Agency for International Development and managed by the Management Sciences for Health, provided comprehensive TB program support in the Amhara and Oromia regions of Ethiopia from 2011 through 2016. Technical and financial support included the preparation of a childhood TB policy, road map, and training manual. The policy and materials were implemented through training of health workers, continuing medical education of clinicians, demonstration of sputum sample collection in children, distribution of childhood TB job aids, supervision, and mentoring.

### Study design and data collection.

We analyzed 3 years of retrospective data for the cohort of TB patients aged 0–14 years who were enrolled in first-line anti-TB treatment during 2012–2014. We extracted relevant data from the health facilities’ TB registers, which include type of health facility, age, gender, TB type, drug dose correctness, HIV test result, and treatment outcome. A semi-structured data collection tool was used to extract patient information from the TB unit registers. Trained health workers with a BSc degree were involved in the data collection.

### Data quality.

Each reporting health facility has a data quality assurance mechanism led by a performance monitoring team responsible for all health-related data.^12^ Data collectors clarified any inconsistency or incompleteness noted during data extraction by consulting the health worker in charge and reconciled the data with the information on the TB patient card kept in the TB clinic.

### Data analysis.

The data collected via the extraction sheet were entered into MS Excel and later imported to SPSS (IBM SPSS Statistics for Windows, version 20, IBM Corp., Armonk, NY) for analysis. Frequencies and proportions were calculated to describe background characteristics and treatment outcomes. Chi-square test and *t*-test for comparison of proportions were computed to compare the association between categorical variables. Logistic regression analysis was used to assess the covariates associated with successful TB treatment. Covariates with *P*-values less than 0.25 in bivariate analysis were included in the logistic regression model. Odds ratios and 95% CIs were used to present the results of logistic regression. *P*-values of less than 0.05 were considered statistically significant.

### Operational definitions.

A bacteriologically confirmed TB case refers to a patient from whom at least one biological specimen is positive for *Mycobacterium TB* using smear microscopy, GeneXpert MTB/RIF (Cepheid, Sunnyvale, CA), or culture.^13^ A clinically diagnosed TB case refers to a patient who does not fulfill the criteria for a bacteriologically confirmed TB case but has been diagnosed with active TB by an experienced clinician who has prescribed a full course of TB treatment. This definition includes cases diagnosed on the basis of X-ray abnormalities or suggestive histology, and extrapulmonary cases diagnosed without confirmation that *Mycobacterium TB* is present.

The standard TB treatment outcome described in the WHO definitions and reporting framework for TB was used (cured, treatment completed, treatment failure, death, lost to follow-up, transferred out, and not evaluated). Treatment success is defined as either a completion of treatment by a TB patient, with smear- or culture-negative results for bacteriologically confirmed cases, or without evidence of failure but with no record to show that sputum smear or culture results in the last month of treatment and on at least one previous occasion were negative, either because tests were not performed or because results are unavailable.^14^ Over- and underdosing for pediatric TB treatment is based on the weight of the child recommended in the national guidelines; deviation from the standard for any dose in the intensive or continuation phase is considered over- or underdosing.^15^

### Ethical considerations.

The Ethical Review Committees of Oromia and Amhara Regional Health Bureaus reviewed and approved the proposed research, including the use of routinely available data and dissemination of the findings. We also obtained permission from the heads of the respective health facilities before extracting the data from TB registers. The data extracted from the registers did not include any personal identifying information.

## RESULTS

A total of 2,557 children (aged 0–14 years) were enrolled for treatment in the 40 hospitals and 137 health centers. One-fourth of the patients were children younger than 5 years (4.7% younger than 1 year and 22.3% aged 1–4 years), whereas 30.4% were aged between 5 and 9 years and the remaining 42.6% were children aged 10–14 years, with a median age (interquartile range) of 8 (4–12) years. The results showed that 853 (33.4) were males, 827 (32.4) females, and 34.2% did not have information about gender on their records. Most of the children, 1,788 (69.9%), were treated in health centers, and the remaining children, 769 (30.1%), were treated at hospitals. The clinically diagnosed TB cases constituted 1,218 (47.6%), extrapulmonary TB 1,100 (43%), and only 227 (8.9%) were bacteriologically confirmed TB (all were from sputum samples); 97.9% were newly diagnosed TB cases; and 88.4% were HIV negative. Appropriate doses of TB drugs were provided to 83.1% and 82.4% of the patients during the intensive and continuation phases, respectively (Table 1).

**Table 1:** Characteristics of children with TB enrolled on treatment

Characteristic	Number (%)
Type of TB	
Bacteriologically confirmed	227 (8.9)
Clinically diagnosed pulmonary	1,218 (47.6)
Extrapulmonary	1,100 (43.0)
No record	12 (0.5)
Category of TB	
New	2,503 (97.9)
Relapse	11 (0.4)
Treatment failure	6 (0.2)
Transferred in	5 (0.2)
Other	17 (0.7)
No record	15 (0.6)
HIV test result	
Reactive	178 (7.0)
Nonreactive	2,261 (88.4)
No record	118 (4.6)
Drug dose, intensive phase	
Appropriate	2,126 (83.1)
Overdosage	121 (4.7)
Underdosage	284 (11.1)
No record	26 (1.0)
Drug dose, continuation phase	
Appropriate	2,108 (82.4)
Overdosage	80 (3.1)
Underdosage	121 (4.7)
No record	248 (9.7)
Treatment outcome	
Cured	175 (6.9)
Treatment completed	2,167 (85.3)
Died	72 (2.8)
Treatment failure	3 (0.1)
Lost to follow-up	28 (1.1)
Transferred out	85 (3.3)
Not evaluated	11 (0.4)

TB = tuberculosis.

The overall TSR was found to be 92.2% (6.9% cured and 85.3% treatment completed). The unsuccessful treatment outcomes included death (2.8%), failure (0.1%), lost to follow-up (1.1%), transferred out (3.3%), and not evaluated (0.4%). The TSRs for the cohort of patients who finished treatment in 2012, 2013, and 2014 showed an increasing trend: 86.9%, 93.3%, and 96.6%, respectively (chi-square for trend = 36.86, *P* < 0.01). A cure rate of 74.4% and a TSR of 93.3% were achieved among bacteriologically confirmed TB cases (Table 1).

The patients who were diagnosed and started treatment in hospitals had relatively lower treatment success than those in health centers (86.5% versus 94.6%, *P* < 0.001). Children younger than 1 year had the lowest TSR (83.5%; 95% CI: 75.4–89.3%), 1–4 years (88.0%; 95% CI: 85.1–90.5%), 5–9 years (93.7%; 95% CI: 91.7–95.2%) and, whereas the 10- to 14-year age-group had the highest TSR (94.1%; *P* < 0.001). The TSR among HIV-positive children was 88.2%, compared with a TSR of 93% in HIV-negative patients (*P* < 0.05). More deaths and loss to follow-up were reported in hospitals than health centers (4.3% versus 2.2% [*P* < 0.01] and 2.6% versus 0.5% [*P* < 0.001], respectively). Similarly, a higher death rate was observed in children younger than 1 year (8.3%), whereas the lowest was reported in the 5- to 9-year age-group (1.9%) (*P* < 0.001). Clinically diagnosed pulmonary TB had a 3.8% mortality (95% CI: 0.7–4.4%) compared with 1.8% (95% CI: 2.8–5.0%) in bacteriologically confirmed TB. The death rate among HIV-positive patients was 8.4%, whereas the corresponding rate among HIV-negative patients was 2.3% (*P* < 0.001). The transferred-out rate was 5.1% in the hospitals as compared with 2.6% in the health centers (*P* < 0.001) (Table 2).

**Table 2:** Tuberculosis treatment outcome by background and clinical characteristics

	Treatment outcome, *n* (%)					
	Successful treatment*	Died	Rx failure	Lost to follow-up	Transferred out	Not evaluated
Region (*N*)						
Amhara (1,285)	1,199 (93.3)	34 (2.6)	1 (0.1)	7 (0.5)	33 (2.6)	11 (0.9)
Oromia (1,256)	1,143 (91.0)	38 (3.0)	2 (0.2)	21 (1.7)	52 (4.1)	0
Type of health facility (*N*)						
Hospital (764)	661 (86.5)	33 (4.3)	2 (0.3)	20 (2.6)	39 (5.1)	9 (1.2)
Health center (1,777)	1,681 (94.6)	39 (2.2)	1 (0.1)	8 (0.5)	46 (2.6)	2 (0.1)
Gender (*N*)						
Male (853)	795 (93.2)	19 (2.2)	2 (0.2)	4 (0.5)	26 (3.0)	7 (0.8)
Female (827)	777 (94.0)	23 (2.8)	0	4 (0.5)	19 (2.3)	4 (0.5)
No record (861)	770 (89.4)	30 (3.5)	1 (0.1)	20 (2.3)	40 (4.6)	0
Age (years) (*N*)						
< 1 (109)	91 (83.5)	9 (8.3)	0	3 (2.8)	5 (4.6)	1 (0.9)
1–4 (569)	501 (88.0)	26 (4.6)	1 (0.2)	10 (1.8)	29 (5.1)	2 (0.4)
5–9 (774)	725 (93.7)	15 (1.9)	0	8 (1.0)	22 (2.8)	4 (0.5)
10–14 (1,089)	1,025 (94.1)	22 (2.0)	2 (0.2)	7 (0.6)	29 (2.7)	4 (0.4)
HIV status (*N*)						
Positive (178)	157 (88.2)	15 (8.4)	0	1 (0.6)	4 (2.2)	1 (0.6)
Negative (2,257)	2,097 (92.9)	51 (2.3)	3 (0.1)	23 (1.0)	74 (3.3)	9 (0.4)
Unknown (106)	88 (83.0)	6 (5.7)	0	4 (3.8)	7 (6.6)	1 (0.9)
Type of TB (*N*)						
Bacteriologically confirmed (227)	212 (93.4)	4 (1.8)	2 (0.9)	4 (1.8)	5 (2.2)	0
Clinically diagnosed pulmonary (1,216)	1,099 (90.4)	46 (3.8)	1 (0.1)	17 (1.4)	51 (4.2)	2 (0.2)
Extrapulmonary (1,098)	1,031 (93.9)	22 (2.0)	0	7 (0.6)	29 (2.6)	9 (0.8)

TB = tuberculosis.

* A sum of those cured and those who completed treatment.

Multivariate analysis showed that belonging to age-groups 5–9 and 10–14 years (adjusted odds ratio [AOR], 95% CI: 2.53, 1.30–4.94; and 2.71, 1.40–5.26, respectively), follow-up at a health center (AOR, 95% CI: 2.51, 1.82–3.48), and HIV-negative status (AOR, 95% CI: 1.77, 1.07–2.93) were significant predictors of successful TB treatment outcomes. Patients with reported drug underdosage in the intensive phase of treatment had a 46% reduction in their treatment success compared with appropriately dosed groups (odds ratio, 95% CI: 0.54, 0.35–0.81). Region and type of TB did not show statistically significant associations with successful TB treatment (Table 3).

**Table 3:** Factors associated with successful TB treatment in children

Variable	Number (*N* = 2,423)	Crude odds ratio (95% CI)	Adjusted odds ratio (95% CI)
Age (years)			
< 1	89	Reference	Reference
1–4	528	1.48 (0.78–2.79)	1.41 (0.74–2.69)
5–9	744	**2.76 (1.45–5.26)**	**2.53 (1.30–4.94)**
10–14	1,062	**3.11 (1.66–5.83)**	**2.71 (1.40–5.26)**
Region			
Amhara	1,254	Reference	Reference
Oromia	1,169	0.74 (0.55–1.01)	1.07 (0.77–1.49)
Type of health facility			
Hospital	737	Reference	Reference
Health center	1,686	**2.72 (2.00–3.69)**	**2.51 (1.82–3.48)**
Type of TB			
Bacteriologically confirmed	219	Reference	Reference
Clinically diagnosed pulmonary	1,146	0.74 (0.42–1.30)	1.09 (0.60–1.98)
Extrapulmonary	1,058	1.18 (0.65–2.11)	1.34 (0.73–2.45)
HIV test result			
Reactive	177	Reference	Reference
Nonreactive	2,246	**1.76 (1.09–2.86)**	**1.77 (1.07–2.93)**
Dose during intensive phase			
Appropriate	2,031	Reference	Reference
High	116	0.86 (0.42–1.73)	0.88 (0.43–1.79)
Low	276	**0.51 (0.34–0.76)**	**0.54 (0.36–0.82)**

TB = tuberculosis. Bold values indicate significant associations.

## DISCUSSION

The TSRs among children with TB who received first-line anti-TB drugs in the study period were higher than others previously reported in Ethiopia.^4,5,9^ Treatment success rates for childhood TB reported in different settings include 45% in Malawi,^16^ 72% in Thailand,^17^ 91.7% in Iran,^18^ and 95% in India.^19^ The TSR in this study is higher than those in reports from Ethiopia in different settings: a TSR of 85.5% in urban health centers in Addis Ababa,^4^ 85.5% in a referral hospital in Addis Ababa,^5^ 83.2% in a referral hospital in southern Ethiopia,^9^ and 66.4% in a rural hospital in southeast Ethiopia.^10^ The proportion of children with bacteriologically confirmed TB, 8.9%, is substantially lower than the corresponding proportion in those aged ≥ 15 years in Ethiopia (27.7%),^20^ but similar to the proportion in children in other reports in Ethiopia.^4^ The observed improvement in TB treatment success might be attributable to ongoing trainings in the regions on comprehensive programmatic and clinical management of TB/HIV, continuing medical education on childhood TB, strengthening of laboratory support, and close mentorship at district health offices as well as health facilities.^21^

We observed an increasing trend in the TSR over the 3-year period, which is probably related to the improvements in TB prevention and control efforts as well as the quality of TB-related health services in the specified period. For bacteriologically confirmed TB cases, the cure rate was found to be 74.4%, which falls within the range of the national target of 72–77% in the specified years.^22^

Hospitals were found to have a relatively lower TSR than health centers. The reasons could be that severely and critically ill patients are referred to and managed in hospitals. Hospitals had more deaths and transfer-outs, which contributed to the lower TSR. Most of the patients had follow-up and adherence support at health centers, serving up to 25,000 people, with the support of satellite health posts, which are located in the community very close to patients. Hospitals need to consider transferring stable patients to health centers for directly observed treatment because the results showed that treatment outcomes in health centers are good, possibly because of lower workloads that facilitated close follow-up. In addition, more patients were lost to follow-up and transferred out in the hospitals, which might have contributed to lower TSRs. Hospitals do not form part of the primary healthcare network in Ethiopia, which works closely with the community via health extension workers (HEWs) to trace children lost to follow-up. Furthermore, hospitals need to devise a mechanism to track the treatment outcomes of transferred-out patients, especially by working closely with the HEWs. The use of digital technology in this regard could be a viable option.^23^ The national childhood TB road map recommends developing integrated family- and community-centered strategies to provide comprehensive and effective services at the community level,^15^ so treatment follow-up and adherence support in health posts for children on TB treatment need to be strengthened.

Treatment success in children undergoing TB treatment depends on the quality of diagnosis and treatment follow-up and of adherence counseling of patients and their families. In our study, underdosage (11.1%) and overdosage (4.7%) were noted in the intensive phase and the continuation phase of treatment (3.1% overdosage and 4.7% underdosage, respectively). Lack of knowledge, failure to monitor patients’ weight, and poor dose adjustment based on children’s weight changes are the likely reasons for inappropriate dosing, which needs further exploration. A multicountry study in Africa reported significantly higher underdosage and overdosage of individual anti-TB drugs than those found in this study.^24^

Overall, deaths, loss to follow-up, and transfer-outs contributed to most of the unfavorable treatment outcomes. More deaths and loss to follow-up were observed in patients followed in hospitals, infants younger than 1 year, and HIV-positive cases. Being older, HIV negative, and followed up in health centers were associated with good treatment success, whereas drug underdosing in the intensive phase was a negative predictor of successful TB treatment. Other factors that play a role in poor treatment outcomes could be delayed TB diagnosis in children, severity of illness, and poor compliance with drug treatment among infants.

Despite the strengths of this study, including reporting previously unavailable evidence on the treatment outcomes of a large number of children with TB from diverse settings, the following limitations should be noted. The data were incomplete because of the retrospective nature of this study, although that issue did not affect the analysis significantly. Some important covariates, such as socioeconomic status, comorbidities, access to health services, health-seeking behavior, and acceptability of the drugs (tablets) to children, were missing because we used the standard national health facility registers devised for routine reporting purposes, which lack those data. A comparison of the result with a baseline was also lacking because there was no nationally disaggregated data on TSRs by age. Another limitation is the exclusion of health facilities with fewer than 10 reported childhood TB patients, because of resource shortages.

## CONCLUSION

The TB TSRs in this study were higher than the national targets for all age-groups as well as the TSRs reported in other studies in the country and the region. Nevertheless, more attention to younger children, hospital settings, and HIV coinfected patients is needed to improve treatment success through better-quality patient care and adherence support and closer monitoring of treatment. Intensive monitoring of children with TB in terms of weight measurement at each follow-up visit, dosage adjustment of anti-TB drugs based on weight changes, and training of health workers on dosage adjustment by child weight could improve treatment outcomes. We recommend a prospective study to understand the contributions to treatment outcomes of social determinants, comorbid conditions, access to health services, new pediatric formulations, and factors related to patient care. We also recommend disaggregation of data by age in the national reporting system.
